# Supplementary material for: Direct imaging of the circular chromosome in a live bacterium
Source: Nat Commun. 2019 May 16;10:2194. doi: 10.1038/s41467-019-10221-0 (PMC6522522; doi:10.1038/s41467-019-10221-0)
Supplement: Supplementary file 1 — Supplementary Information [file 41467_2019_10221_MOESM1_ESM.pdf]

Supplementary Information

Direct imaging of the circular chromosome in a live bacterium

Fabai Wu[1,2*], Aleksandre Japaridze[1*], Xuan Zheng[1], Jakub Wiktor[1], Jacob W. J. Kerssemakers[1] & Cees Dekker[1,#]

1. Department of Bionanoscience, Kavli Institute of Nanoscience Delft, Delft University of Technology, Van der Maasweg 9, 2629 HZ, Delft, The Netherlands
2. Division of Geological and Planetary Sciences, California Institute of Technology, 1200 E California Blvd, Pasadena, CA 91125, USA
[*]These authors contributed equally.
[#]Correspondence: c.dekker@tudelft.nl

This SI includes:

- SI Methods
- Supplementary Figures 1-19.
- Supplementary Table 1. List of strains used in this study.
- SI References

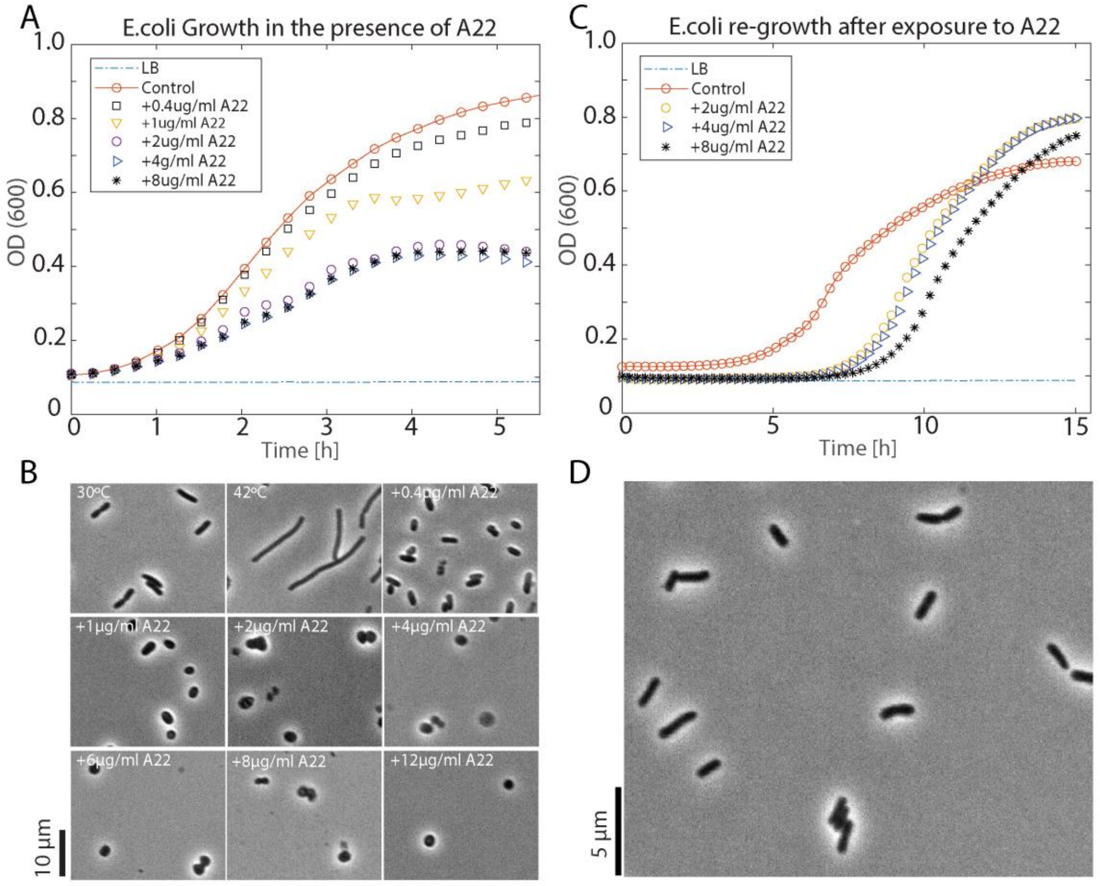

Supplementary Figure 1. Cell growth curves in the presence of A22 drug.
A. Cell growth curves (measured by the optical density at 600nm in LB at 30ºC) in the presence of various concentrations of A22 drug (0.4µg/ml - 8µg/ml). Cells are clearly alive during time of the experiment (5 hours after incubation), even as the higher drug concentrations partially slow down the growth.
B. Phase contrast images of cells grown on LB after 5 hours of exposure to the A22 drug. Cells grown at 30ºC without A22 maintain typical rod shapes. Cells grown at 42ºC elongate and become significantly longer in size. In the presence of A22, cells adopt round shapes for A22 concentrations higher than 2µg/ml. A22 drug concentrations used were 0.4µg/ml - 12µg/ml. Scale bar, 10 microns.
C. If the A22 drug is removed after 4 hours of exposure at 40ºC (as in the microscopy experiments), the cells re-start growth.
D. After the A22 drug is removed, the cells re-cover the rod shape. The image show cells which were exposed to 4µg/ml A22 for 4 hours at 40ºC and then transferred to LB at 30ºC. This image was taken 24 hours thereafter and shows that these cells recovered rod shapes. Scale bar, 5 microns.

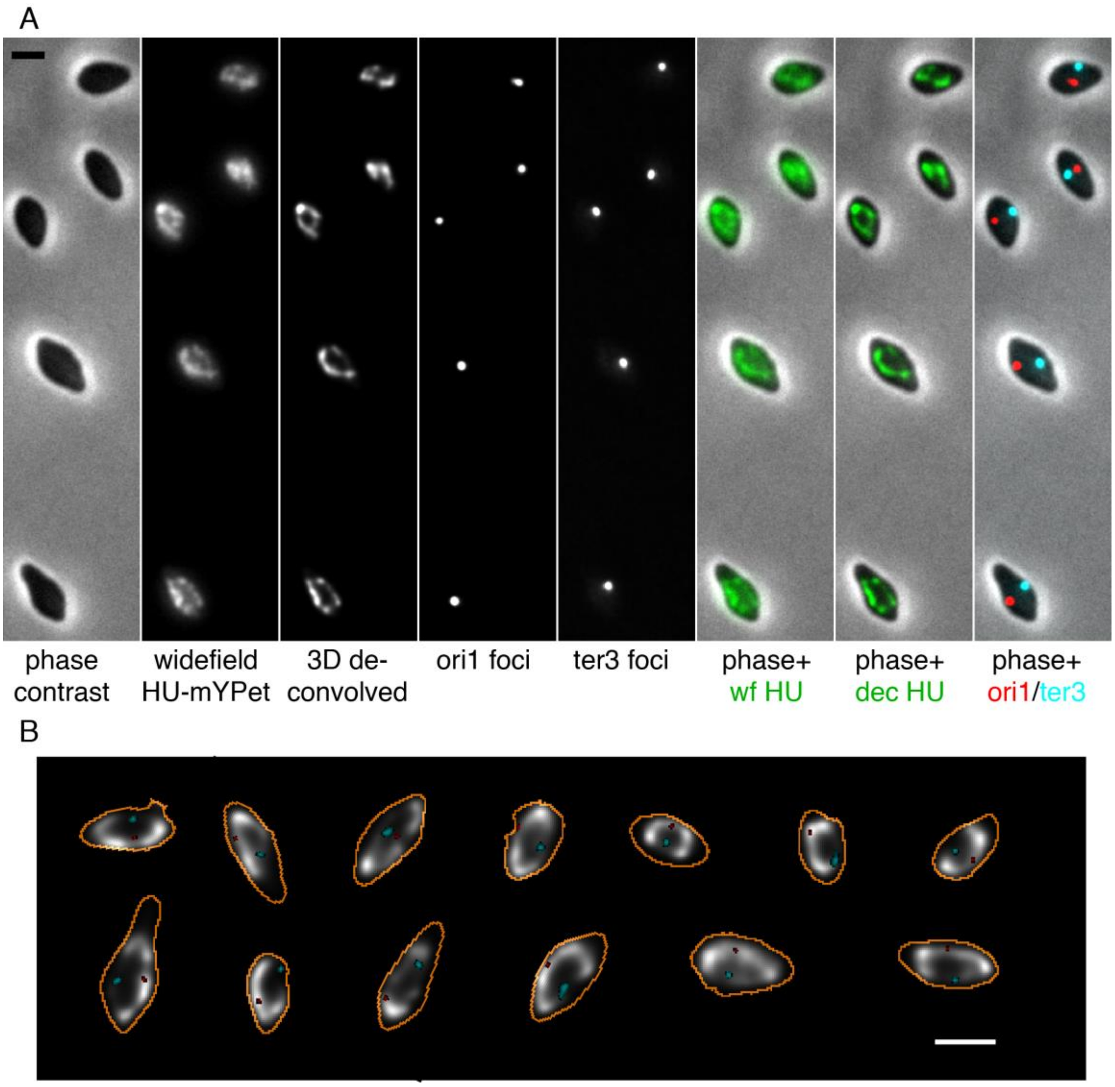

Supplementary Figure 2. Circular chromosomes visualized in widened cells.
A. Widened cells in the same field of view imaged with phase contrast and different fluorescence channels, before and after deconvolution.
B. False-color overlay of more examples of single cells with HU-mYPet in grey scale, Ter3 foci in cyan, Ori1 foci in red and cell boundary in orange.
Scale bars, 2 microns.

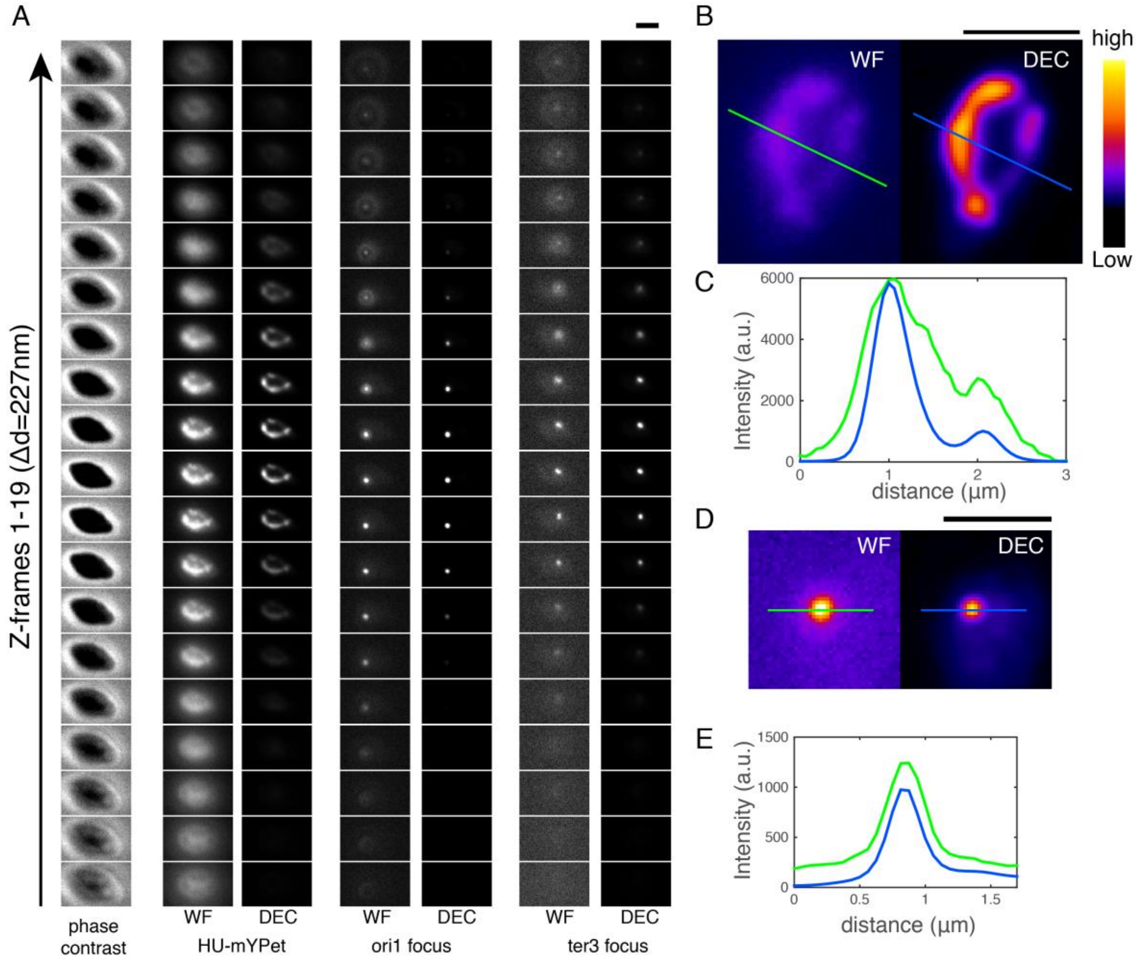

Supplementary Figure 3. 3D deconvolution to reduce out-of-focus noise.

A. Original widefield images and 3D-deconvolved images in different fluorescence channels.

B. Images comparing the Z-stack-midframe original and deconvolved images in the YFP channel (HU-mYPet). Color bar indicates intensity.

C. Fluorescence intensity profile across the lines in B.

D. Images comparing the midframe original and deconvolved images in the RFP channel showing the Ori1 focus.

E. Fluorescence intensity profile across the lines in d. Note that the left side of the profile indicate space outside of the cell, where the background was identified and reduced to zero through deconvolution, whereas the right side is the cellular space, in which freely floating LacI-mCherry proteins result in nonzero background.

Scale bars, 2 µm.

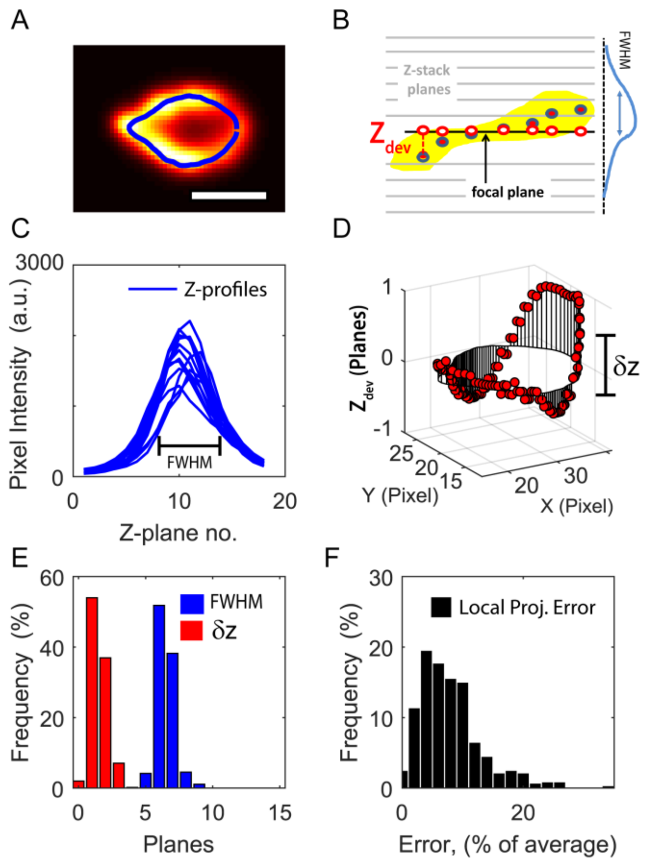

Supplementary Figure 4. Effects of approximating the 3D chromosome structure by a 2D projection.

A. Typical example of a deconvolved image of a circular chromosome in the focal plane. The blue line depicts the ‘backbone ridge’ along which the DNA density is measured. Scale bar 1 µm.

B. Schematic side view of chromosome geometry (XZ plane). For the analysis such as in panel A, we use a single focal plane (white markers). If the local chromosome mass (yellow) is tilted or partly out of the focal plane, the measured intensity in the focal plane will be lower than the real intensity associated with its local center of mass. The blue line depicts a schematic Z-profile through the rightmost marker. Note that the larger the full width half maximum (FWHM) of such a profile, the smaller the relative difference between the true intensity and that measured at the focal plane.

C. Signal intensity versus Z-plane coordinate measured at various points along the contour line in panel A. Z planes were acquired every 227 nm. The FWHM of each profile (typically ~6 planes) indicates the effective focal depth.

D. 3D depiction of the donut backbone contour which tracks the maximum density along the circular chromosome, taken from the Z-positions of the profiles shown in panel C plotted along the contour line in panel A. Note the flatness of the structure (please note that the vertical scale is strongly exaggerated).

E. Histogram of the vertical spread per cell (red bars) for N=100 cells compared to the FWHM width of the Z-profiles (blue bars). The spread is defined as the standard deviation (one sigma) of vertical positions per cell (cf. δz in panel D). Clearly, the majority of z-positions along a donut falls well within the vertical focal range of the microscope, implying that curving up or down of the donut structure will not change the measured density much. The average standard deviation from midplane position is 1.5 Z-planes (335nm).

F. Local deviation of the focal plane intensity from the actual maximum intensity (as schematized in B) along the chromosomal contour, deduced for N=100 cells. Typical differences are of the order of 5-10%.

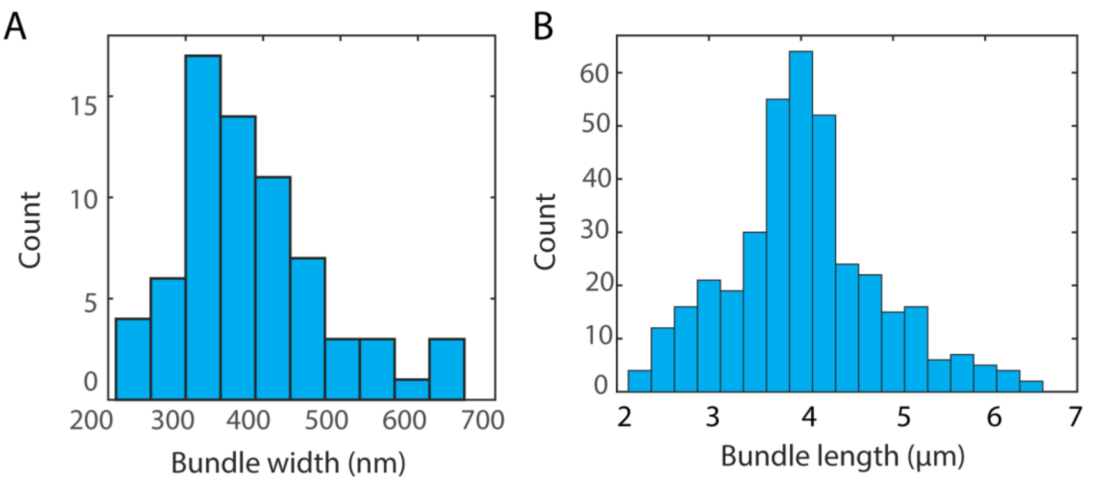

Supplementary Figure 5. Time-average values of chromosome width and contour length distribution measured by 2D-SIM images.

A. Average chromosome bundle widths quantified as the full-width-at-half-maximum of the peak intensity across the donut chromosomes (width = 0.40 ± 0.02 µm) measured with SIM (N=69).

B. Histogram of chromosome bundle lengths (L=4.0 ± 0.8 µm) measured with SIM (N=374)

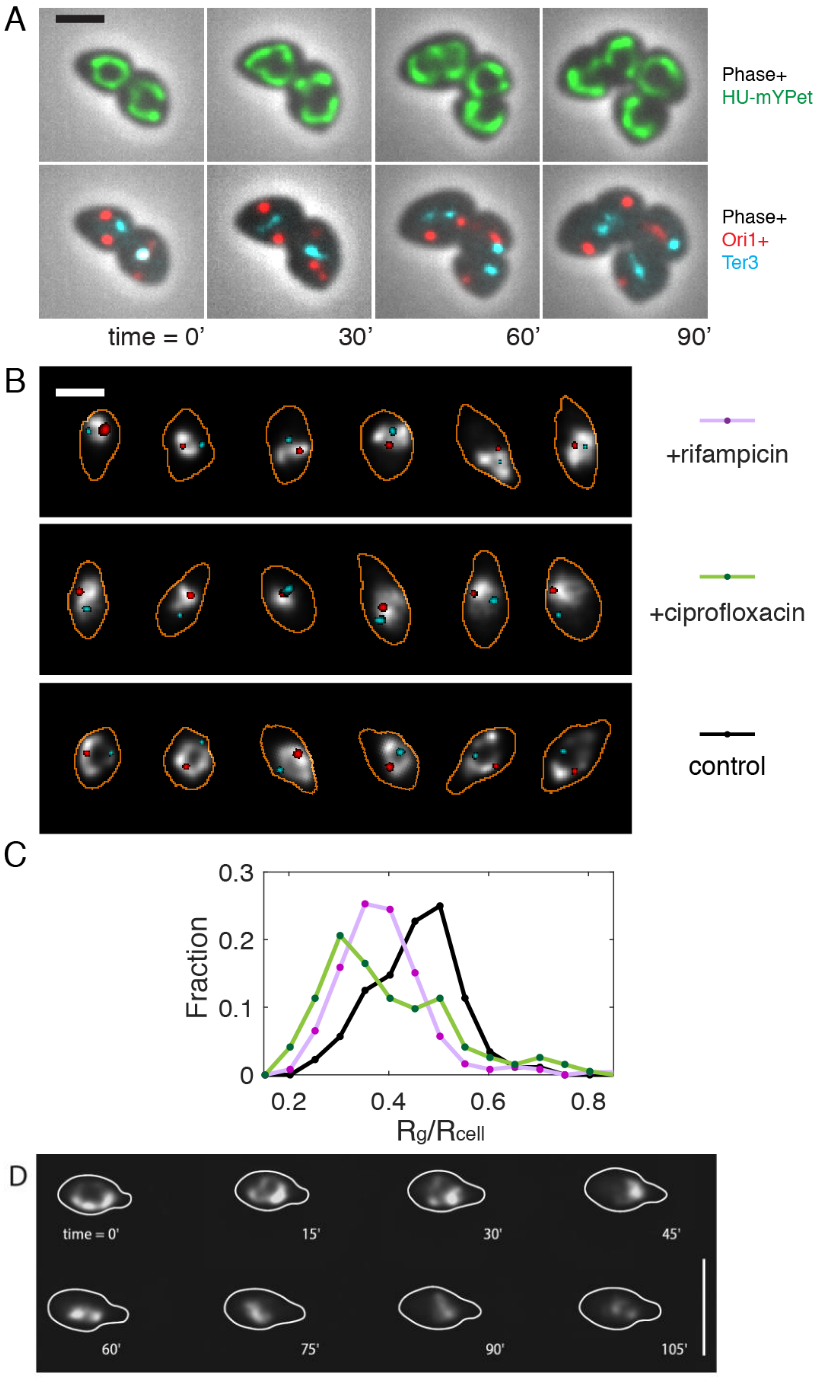

Supplementary Figure 6. Circular chromosomes support replication and are perturbed by antibiotics.
A. Wide-field time-lapse images showing the replication of circular chromosomes (green) and the division of the corresponding cells (phase contrast images in grey scale).
B. Example 3D-deconvolved images of wildtype chromosomes (control) and those treated by ciprofloxacin, rifampicin. Cell boundary in orange, HU-mYPet signal in gray scale; Ori1 foci in red; Ter3 foci in cyan.
C. PDF of the ratio of the radius of gyration for a chromosome and that of a cell, in widened cells with and without antibiotics treatment (same color code as panel B).
Scale bars, 2 μm.
D. Time-lapse fluorescent images showing the collapse of the toroidal chromosome structure into a condensed blob upon a sudden shift to stationary phase. White outlines the cell boundary, scale bar, 5 μm.

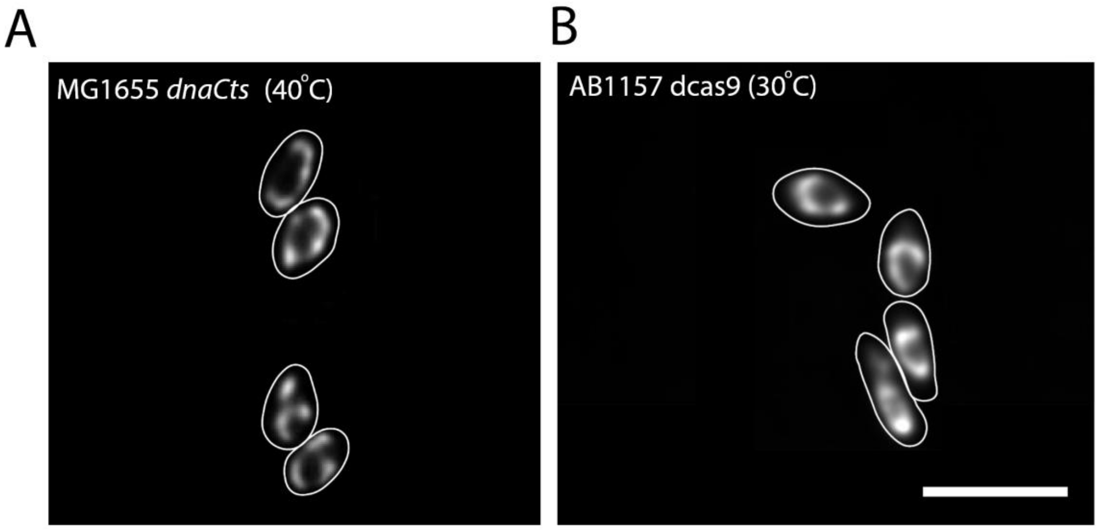

Supplementary Figure 7. Torus chromosomes is a general feature in re-shaped *E.coli* cells with a single chromosome, irrespective of strain type.
3D-deconvolved images in the YFP channel (HU-mYPet) showing heterogeneous circular chromosomes in **A.** MG1655 *dnaCts* cells and in **B.** AB1157 cells with dcas9 blocked replication indicating that the torus topology is a general feature of the chromosome and does not depend on the *E.coli* strain used, neither on elevated temperatures to maintain a single chromosome in temperature sensitive cells (*dnaCts*). White outlines the cell boundary, scale bars, 5 μm.

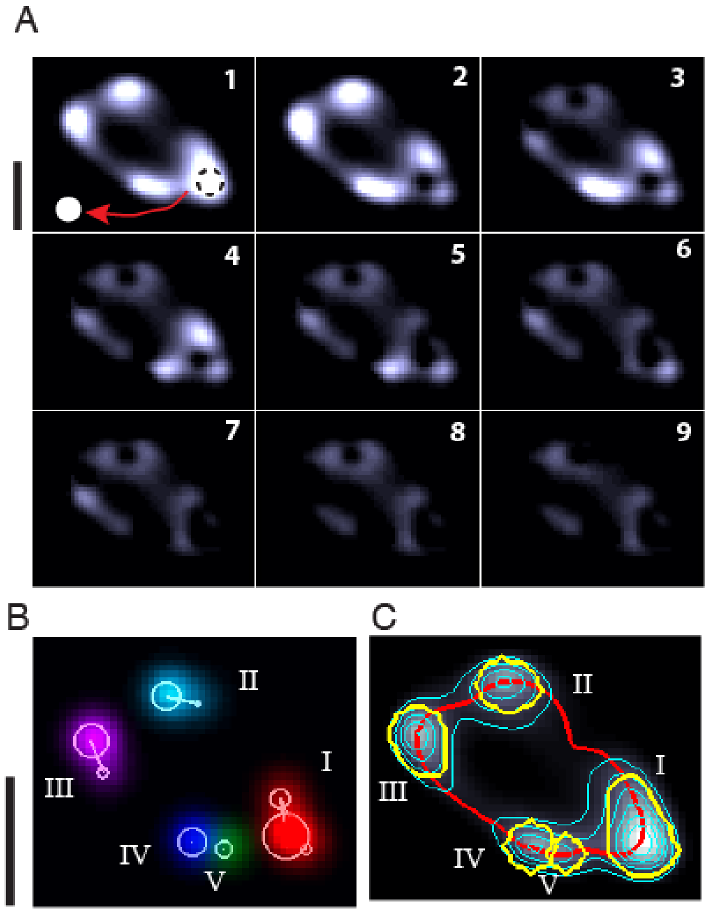

Supplementary Figure 8. Automated cluster analysis.

A. Example of decomposing a circular chromosome pattern (see also Main text, Fig.2a). The image is decomposed by peeling off of the brightest peak (red arrow in panel 1) by a single-spot that is a point-spread-function (PSF) limited Gaussian (size indication in inset). This procedure is repeated on the resulting image (panel 2) and continued until only a small residual intensity remains (panels 3-9), where all intensity is below a set threshold. Typically, this routine needs ~10 spot subtractions. We note these spots are not interpreted as single dye spots, but just as 'components' of an otherwise complicated image pattern. Note that the intensity of the image is scaled to oversaturation for the ease of visualization of the weaker intensity parts.

B. Peeled-off spots are collected (circles) and are joined into groups when they are separated by a distance smaller than one PSF width. Adding up such adjacent spots per group re-creates separate clusters, labelled I-V in the example (indicated in different colours). Brighter spots are represented by larger circles (while each spot has the same FWHM set by the PSF).

C. For further processing, each reconstructed cluster, can be analysed for relative content, size, position etc (see Main Text). For example, yellow lines define clusters contours at an intensity level of 20% of the brightest part of the chromosome. In addition, cluster positions can be related to global genomic position by combining the result with the ridge loop analysis (red line). Scale bars, 2μm.

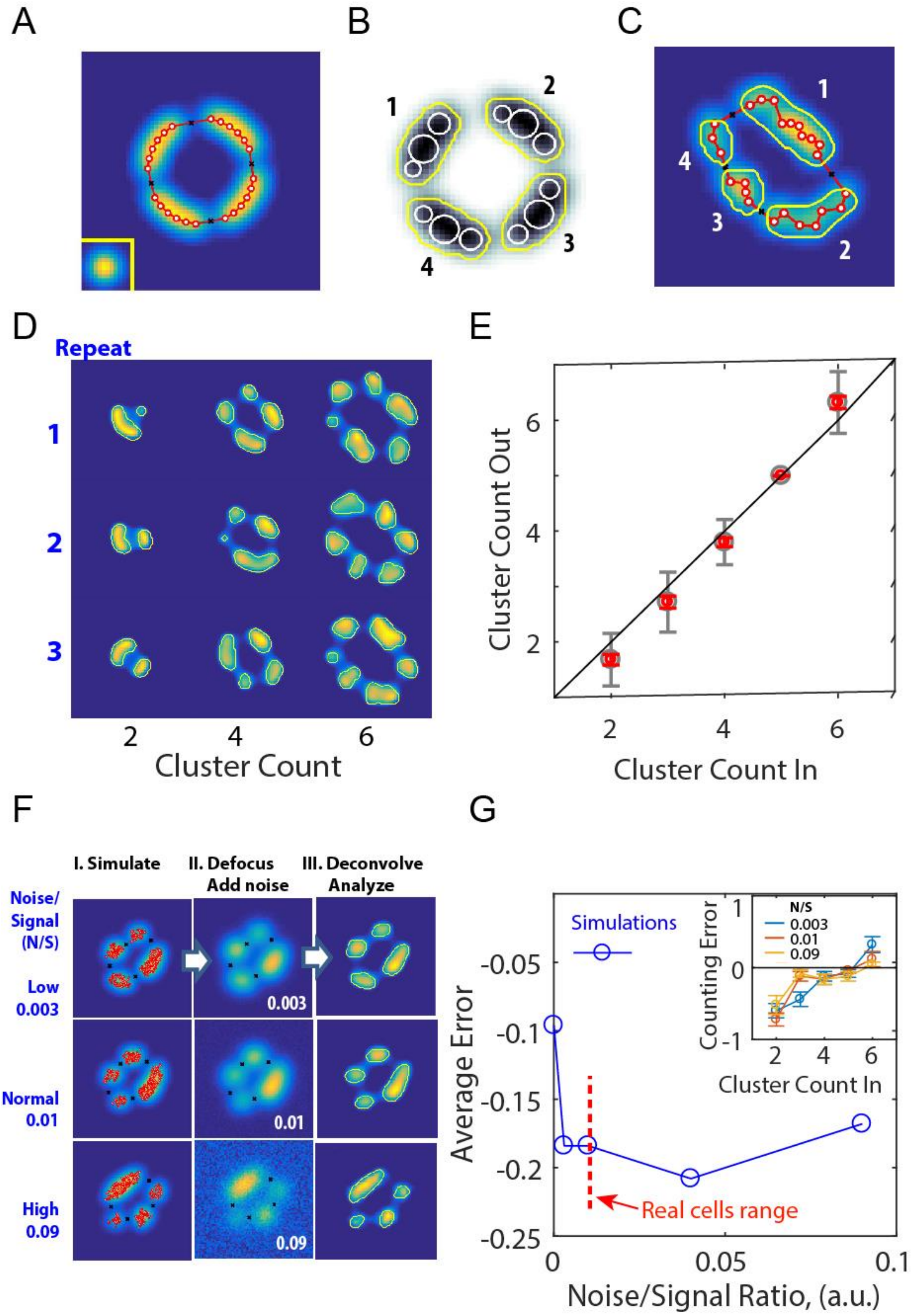

Supplementary Figure 9. **Performance tests of the cluster analysis.**
A. Modeling a group of equal-size blobs in a donut configuration. Each blob consists of five Gaussian spots that are mutually spaced within one point spread function (PSF, see inset) from each other to create a smooth structure. Groups are mutually separated by a spacing larger than 1 PSF. Convoluting the pattern results in a well-defined structure made up of four equal-size clusters separated by gaps (black crosses).

B. Result of our cluster analysis of the pattern in (A). Note that the aim is not to reconstruct the originally single-Gaussian spots that were merely a tool to generate the clusters, but to provide an efficient means of describing each cluster by a low number of single Gaussian components of varying amplitudes. The result of the cluster analysis is 4 clusters (denoted by the yellow contour lines) that trace the input pattern A well. The white circles denote the individual Gaussian spots used for describing the clusters.
C. To more closely resemble the experimental nucleoids, the sizes of the clusters were randomized. The yellow contour line indicates the result of the cluster analysis performed on an artificial, irregular pattern (white markers) built of closely spaced Gaussian spots separated by gaps (black crosses).
D. Series of 2/4/6 simulated clusters, each with 3 repeats, showing the random variability of cluster size and shape.
E. To quantitatively evaluate the effect of randomized positions, we examined the effectiveness of the cluster detection protocol on such random patterns. The graph shows the count of the number of clusters that was found by the cluster-analysis routine vs. the input number, for irregular patterns such as shown in panel D. Each data point shows the average, the standard error of the mean (red error bars, N=100), and the standard deviation (grey error bars) per set of cluster number. An excellent tracing is performed for cluster numbers of 3 to 5, while small deviations are seen at lower and higher numbers.
F. Estimation of the effect of background noise on the cluster analysis. Left panels: clusters that are shaped into irregular, randomly sized patterns using large numbers of closely spaced spots separated by gaps. Middle panels: Same images, transformed by a defocus scheme estimated by the defocusing behavior of small beads of 0.2 μm diameter (Invitrogen, TetraSpek blue/green/red microspheres). Furthermore, noise is added to varying degrees. We define a noise-to-signal ratio (N/S) where noise N is the RMS background intensity away from the chromosome, and signal S is the average intensity value at the chromosome peak contour. Shown, from top to bottom, are typical examples for ‘low’, ‘normal’ and ‘high’ N/S ratios respectively. The ‘normal’ N/S ratio is comparable with the values measured for experimental images (see panel G). Right panels: mimicking the real acquisition process, these images are subsequently deconvolved and cluster analyzed.
G. Effect of background noise on the accuracy of the cluster analysis. Shown is the average counting error that was observed in the cluster count as averaged over all cluster numbers (2-6), cf. inset, as a function of N/S ratios. For real cells in our experiments, we find an N/S ratio of 0.013 ± 0.003, (N>100) as indicated by the red dashed line. For realistic noise levels, the average counting error is only of order 0.2 counts. Inset: Average counting error (N=25) as a function of cluster number for the three different noise levels shown in panel F.

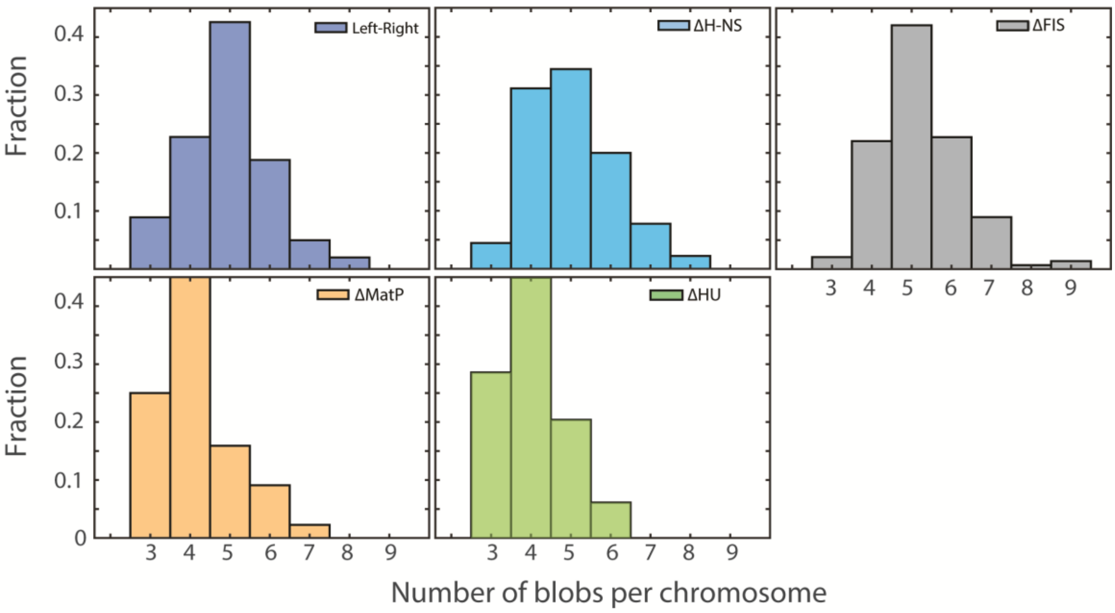

Supplementary Figure 10. Number of blobs per chromosome for various chromosome labels and mutants.

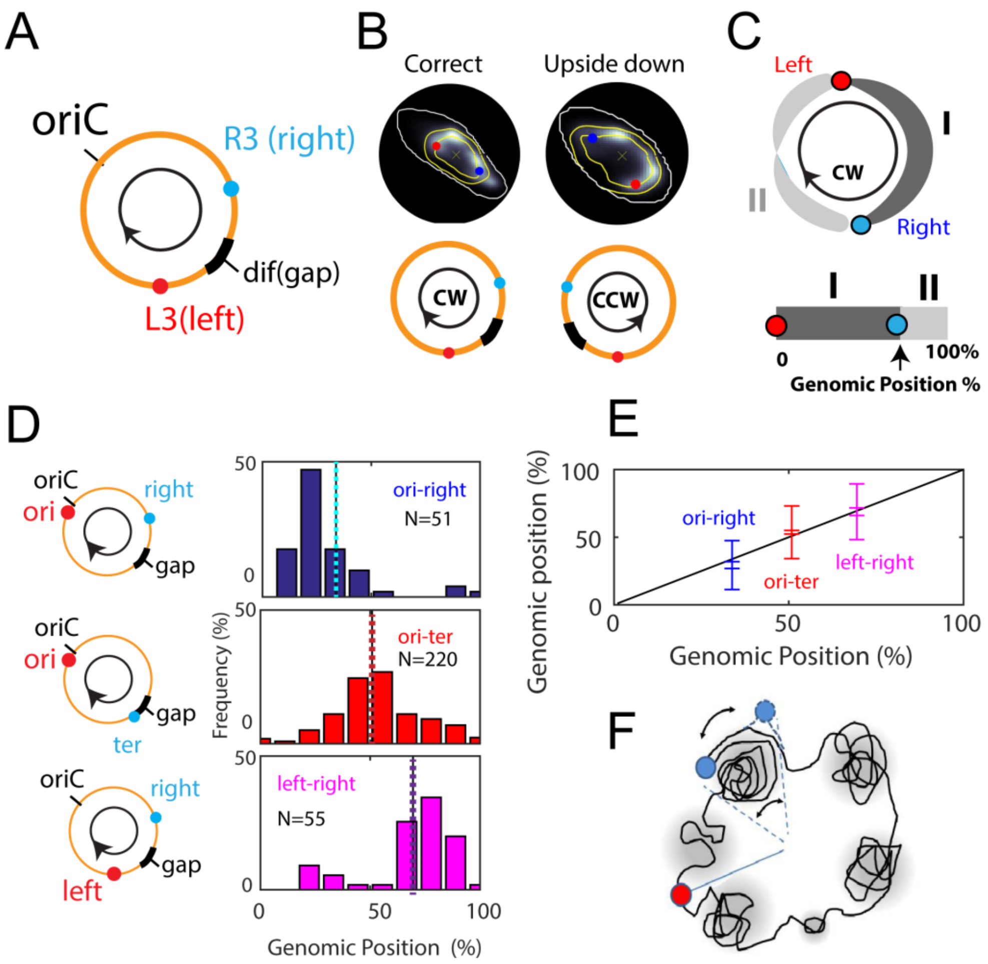

Supplementary Figure 11. Estimating the genomic position based on DNA fluorescent intensity.

(A) Example of expected genomic positions and gap region, in this example, for *E.coli* with FROS tags in the left and right chromosome arms.

(B) Deconvolved fluorescence images (top) of the two possible imaging orientations of the circular genome. Bottom: based on the relative order of label 1, label 2, and the gap region, the orientation can be established, and with that, the global sequence direction (arrows) are identified as clockwise (CW) or counterclockwise (CCW).

(C) Following the sequence direction in (B), the fluorescence intensity (here measured CW, starting at the left arm (red circle) between the labels serves as a measure for the genomic content between these labels. This content can then be translated to a genomic percentage-of-genome axis (bar at the bottom).

(D) Histograms of the measured genomic distances for three different strains with different sets of FROS tags : (top) ori-right (genome ratio: 34:66%), (middle) ori-ter (genome ratio: 51:49%) and (bottom) left-right (genome ratio: 70:30%) FROS arrays. Dotted lines denote the expected position of the tags.

(E) Comparison of measured mean genomic positions to the known positions of the labels shown in (D). Small error bars indicate SEM, large bars indicate SD (+/- 1 sigma). On average, the labels are found to be located at the expected positions. Around this average position, we observe a cell-to-cell spread of about 15% (1 SD).

(F) Schematic depiction of the possible source for the observed spread of positions: small rearrangements within a cluster (grey area) can cause a positional fluctuation over a distance of maximum twice the cluster size. With a typical cluster size that amounts to about 15% of the genome size, such deviations explain the observed spread.

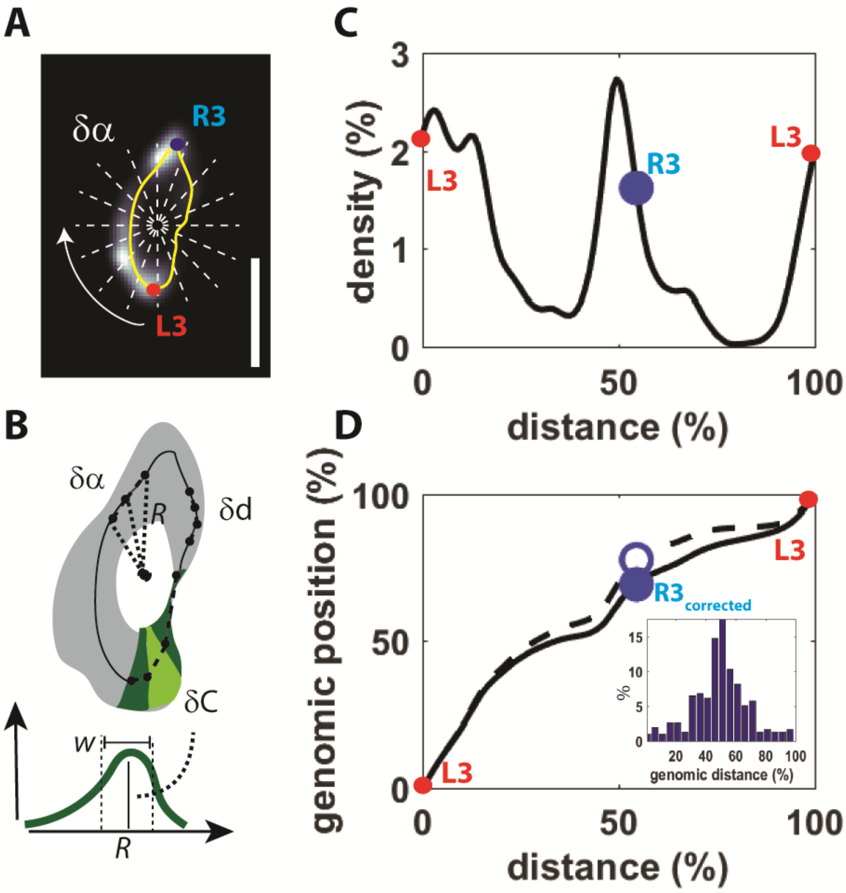

Supplementary Figure 12. Automated deduction of the chromosome ridge line and density distributions.

A. Example of a chromosome with L3/R3 labels (see also Fig.3b in the main text). Angular sections (dashed lines) are defined from the centre-of-mass of the chromatin pattern. For illustrative clarity, 16 sections are shown here, whereas in practice we analysed the data using 100 sections. Scale bar, 2μm. Additional labels at the L3 (red) and R3 (blue) positions serve to determine the global genomic order of these angular sections.

B. Each angular section yields a radial profile (top left) from which local properties such as section content δC, bundle width w and radial peak position R are derived for every angular step $\delta\alpha$. For further processing, the values are re-sampled at equidistant (δd) points (top right) along the line following the ridge line, i.e. the thin solid line. This 'ridge loop' forms the main axis for further analysis. The shaded green areas indicate areas of equal fluorescence content δC (bottom), which can be used to reconstruct a global genomic axis.

C. Example of a 'DNA density curve' for the chromosome in panel A, showing the local DNA content vs. distance along the ridge line, starting at *L3*, both in percentage of the total. The blue marker indicates the distance and local density associated with the R3 label.

D. Genomic position as function of distance. The dashed curve shows the data as-measured. The corrected curve (solid black line) uses the additional experimental information (as we do know the genomic position of the R3 label) to enforce the correct R3 label genomic position (blue). The figure inset shows the histogram depicting positions of ter foci relative to the genomic distance. As expected, we see a peak near 50% (the expected position) with the majority of ter positions being within ±15% from the expected position. Here, ±15% is the largest error that the foci position can have, which roughly corresponds to the size of the DNA stored in a typical cluster.

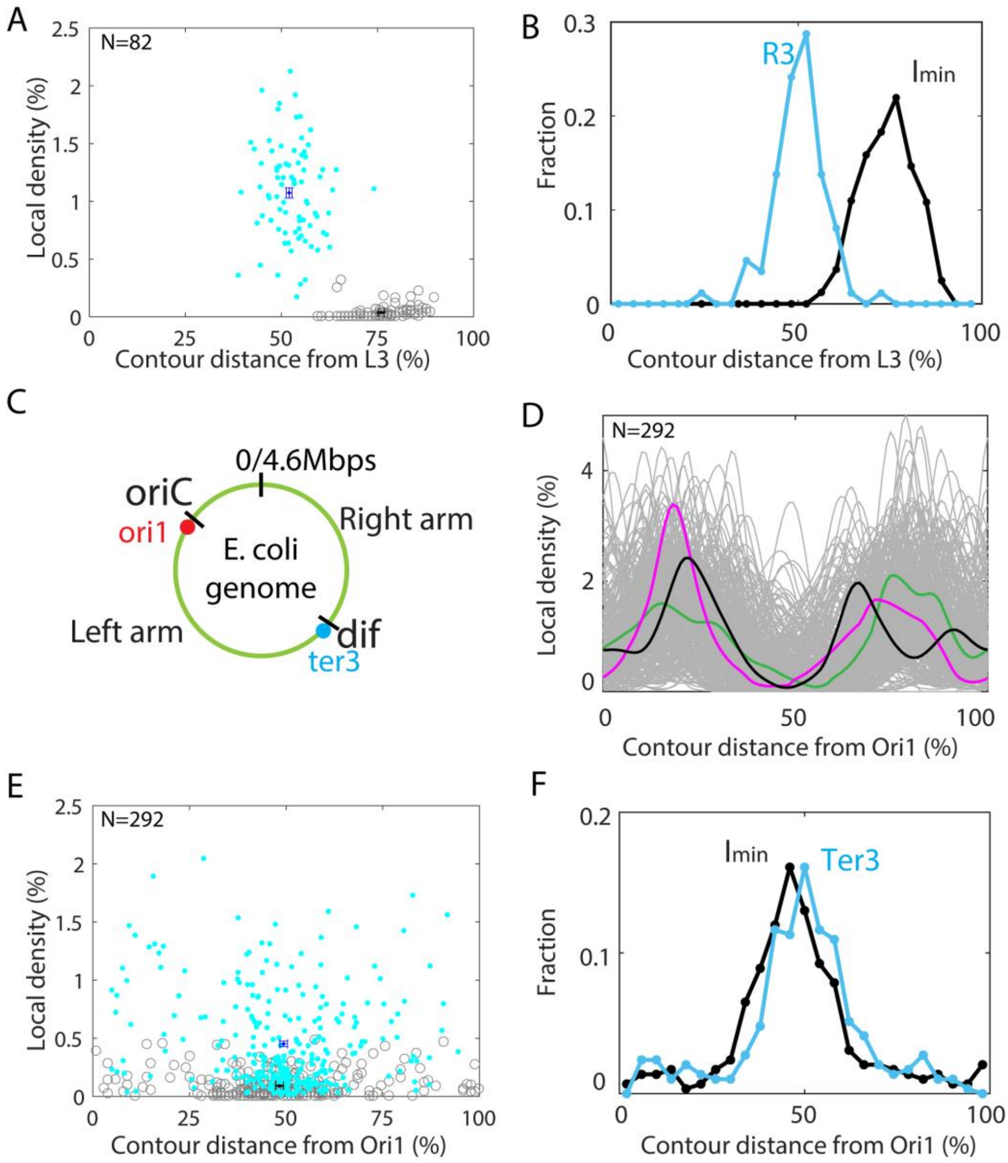

Supplementary Figure 13. DNA density and localization of various genomic foci and global minima.

A. DNA density of the R3 loci (cyan dots) and global minima (grey circles) in a strain with L3/R3 labels. Mean ± standard errors were also plotted in blue and black, respectively.
B. PDF showing the distance (measured in % of the total chromosome length) of the R3 foci and the global density minima from the L3 foci along the contour.
C. Illustration of a chromosome with the Ori1 and Ter3 labels for d-f. Note that the two branches separated by these two labels are almost equal in length, and as a result the left and right arms cannot be well distinguished for this strain.
D. Local DNA density along the ridge lines of single circular chromosomes in a strain with Ori1/Ter3 labels. Three random examples are highlighted to illustrate the heterogeneity.
E. DNA density of the Ter foci (cyan dots) and global minima (grey circles) in a strain with Ori1/Ter3 labels. Mean ± standard errors were also plotted in blue and black, respectively. Localization is indicated by the distance (% total length) along the contour from the Ori1 loci.
F. PDF showing the distance (% total chromosome length) of the Ter3 loci and the global density minima from the Ori foci along the contour.

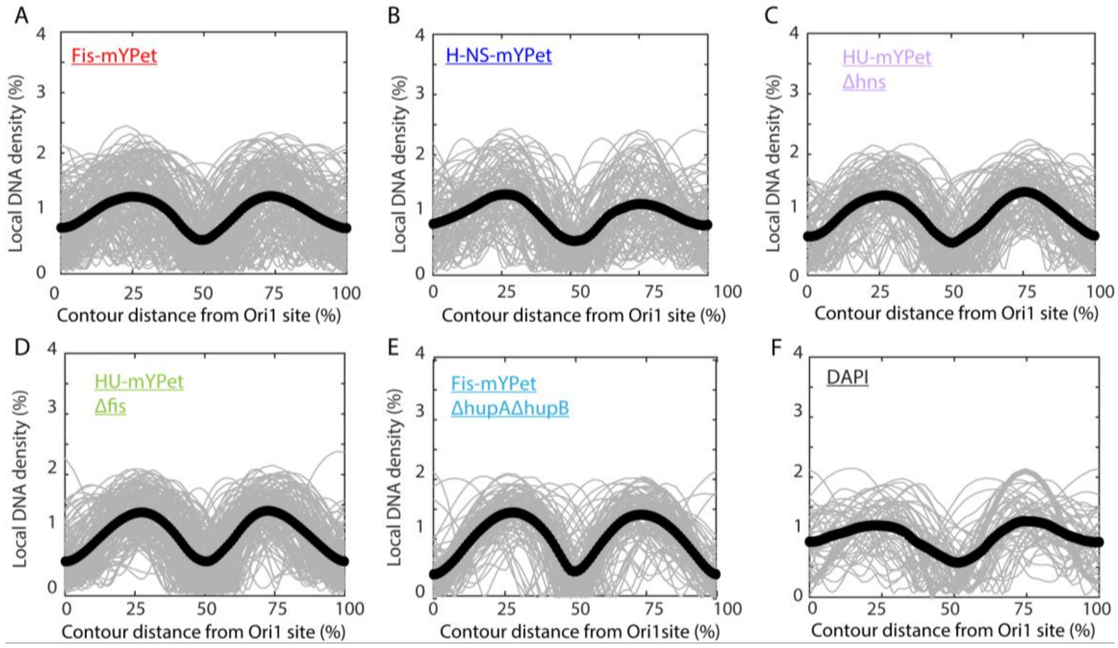

Supplementary Figure 14. M-shaped DNA Density distributions along the chromosomal torus for strains with different labels and NAP deletions.

A-F. Single cell data in various strains. The number of cells are respectively N=152 (A), 92 (B), 90 (C), 149 (D), 98 (E), 47 (F). Note that all strains are labeled at the Ori1 and Ter3 loci.

F. Average density distribution from Ori1 to Ter3, with the two arms folded. The colors correspond to the strain descriptions in A-E. Black indicates strain with HU-mYPet label as shown in Fig. 3F and Fig. S7D.

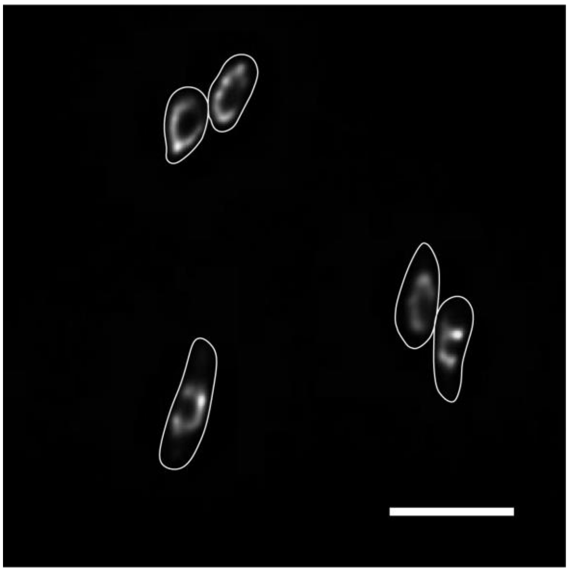

Supplementary Figure 15. Circular chromosomes in AB1157 dnaCts strain without tetO and lacO repeats.
Deconvolved images in the YFP channel (HU-mYPet) showing heterogeneous circular chromosomes, white outlines the cell boundary. Scale bars, 5 μm.

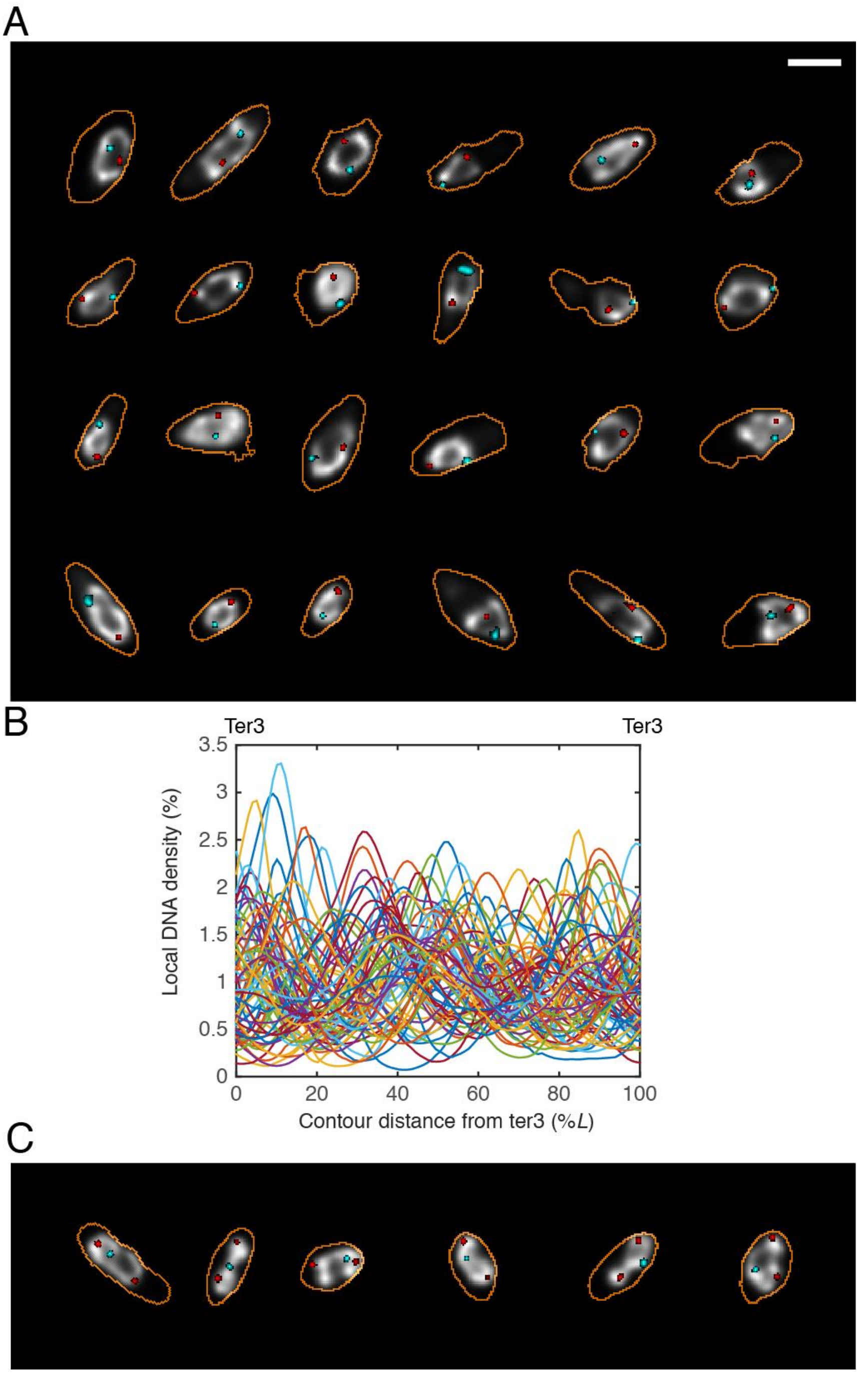

Supplementary Figure 16. Chromosome morphology and DNA density distribution for *ΔmatP* cells.
A. Deconvolved images of circular chromosomes in *ΔmapP* cells. Note that in most cells no apparent gap exists near the Ter3 foci, in contrast to in WT cells, cf. Fig. 1 and Fig. S1. Orange outlines the cell boundary, HU-mYPet is in grey scale, red indicates Ori1 foci, cyan indicates Ter3 loci. Scale bar, 2 µm.
B. Local DNA density along the ridge lines of single circular chromosomes in the *ΔmapP* strain with Ori1/Ter3 labels. Position of Ter3 and average position of Ori1 loci are indicated.
C. Replicating chromosomes of *ΔmapP* cells (as shown in a) after a brief restart of replication (deconvolved imges).

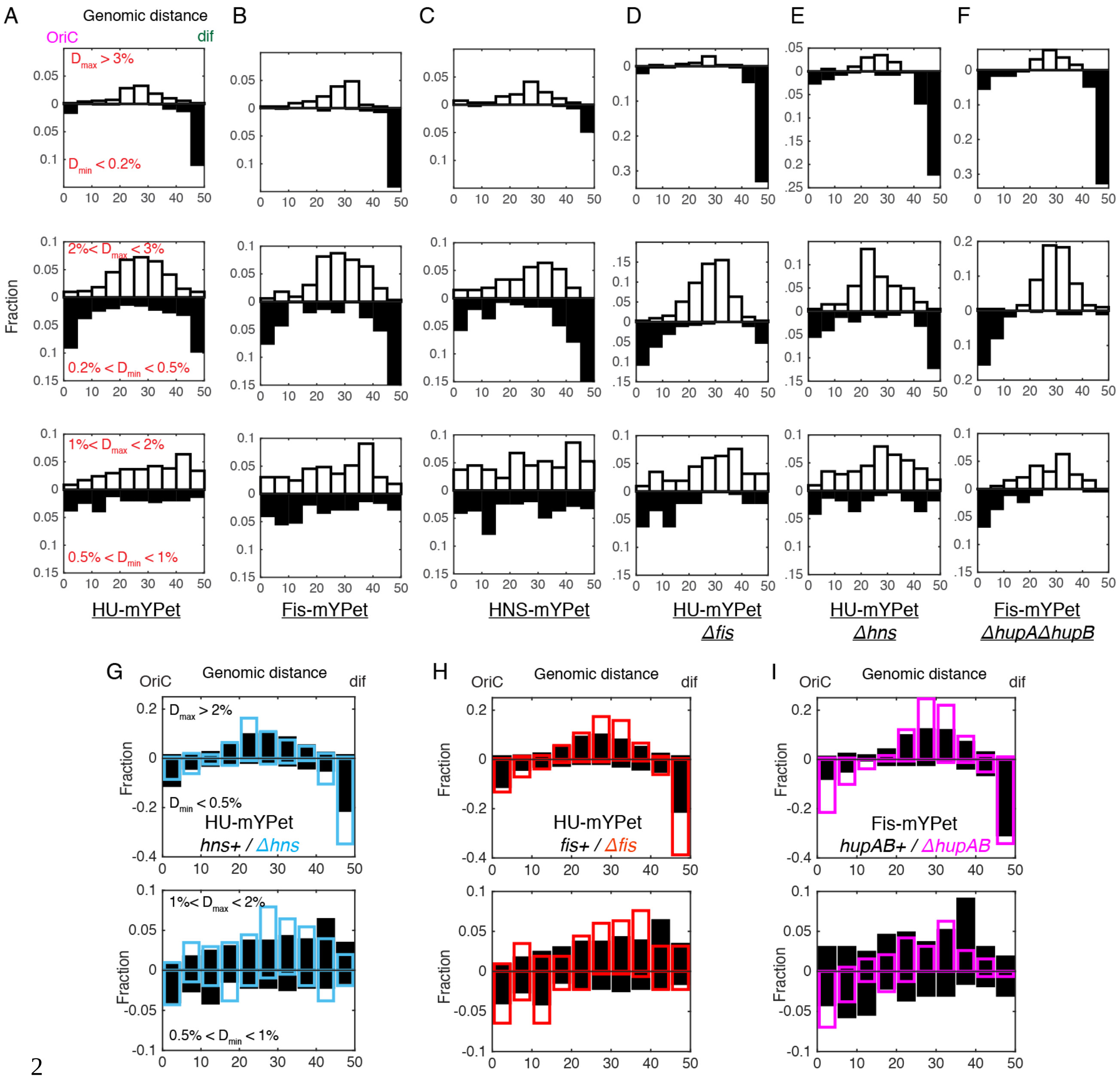

Supplementary Figure 17. Probability density function comparing the distributions of local maxima and local minima of DNA intensity in wildtype and NAP-mutant strains.
A-F. Each panel shows the frequency of local maxima (plotted upwards) and local minima (downwards) for, from top to bottom, three different levels of density. The fluorescent labels and mutations are shown at the bottom. Density levels are all as indicated in A.
G-I. Comparisons between wildtype and three different NAP deletion mutants. Density levels are all as indicated in g.

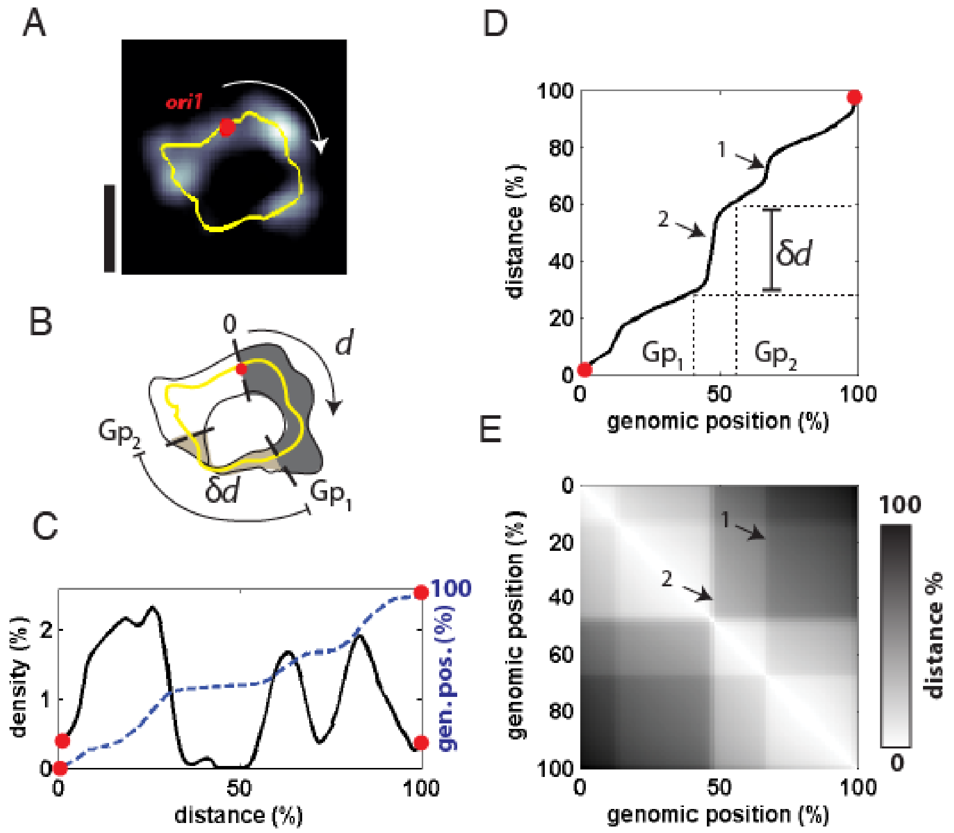

Supplementary Figure 18. Automated proximity map construction

A. SIM microscopy image of a chromosome with the *ori1* (red) and ridge line (yellow) indicated. Scale bar, 2μm.

B. Starting at ori1 as the zero point, distance *d* is measured clockwise along the contour (top right arrow). The summed intensity (light-grey shaded area) is a measure for the relative genomic positions Gp_1 and Gp_2. A distance δd can be defined from measuring the physical distance between these positions along the ridge line.

C. The associated local genomic density (black) is measured along the entire torus and integrated to obtain the genomic position (dashed blue).

D. Next, the data is interpolated to plot the inverse, viz., the distance as a function of genomic position. From this curve, the relative distance δd between any two genomic positions Gp1 and Gp2 can be deduced.

E. The distances thus deduced between any two points Gp_1 and Gp_2 define a two-dimensional 'proximity map', where white, square areas denote domains of genomic material that is closely adjacent in physical space.

The numbered arrows in (D) and (E) point to corresponding features.

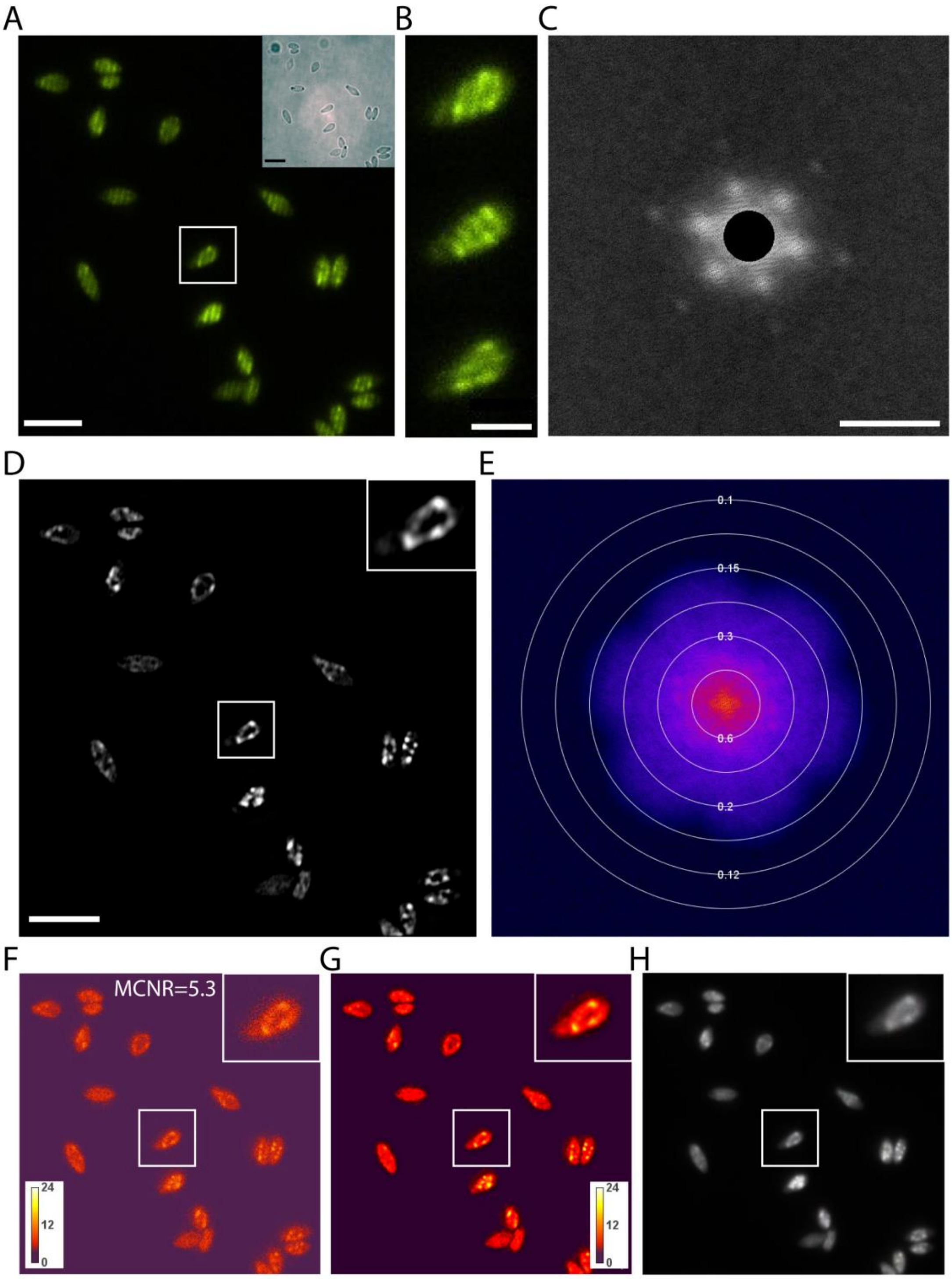

Supplementary Figure 19. SIM imaging controls

SIM images were tested for possible imaging and reconstruction artefacts, using the SIMcheck software (1).

A. Typical example of a raw SIM image of widened *E.coli* cells with HU-mYpet labelling. Inset shows a brightfield image of the same sample. Scale bars, 5 μm.

B. Zoomed image of the cell highlighted in panel A. The dark grid-like modulation pattern in the three images shows each of the three illumination angles. Scale bar, 2 μm.

C. Raw Fourier Projection of the raw data in reciprocal space, with points of high-frequency information from first (large inner spots) and second (outer smaller spots) order stripes. Scale bar 16 μm^{-1}.

D. Reconstructed SIM image from the image in panel A. Inset shows a zoomed image of the circular chromosome from panel B. Scale bar, 5 μm.
E. Reconstructed Fourier Plots overlaid with concentric rings that indicate the corresponding spatial resolution in micrometers. Based on the profiles one can approximate the effective resolution limit of features on the reconstructed data to be ~ 0.15μm.
F & G. Modulation Contrast Maps for Raw (panel F) and reconstructed SIM images (panel G). The higher the value of the modulation contrast-to-the noise ratio (MCNR) (i.e., the higher the yellow-to-red color intensity) the higher the quality of SIM reconstruction. The modulation contrast-to-noise ratio (MCNR) for the full raw image was equal to 5.3, which is a satisfactory value (1), also considering that we image live bacteria and not fixed cells (1).
H. Motion & Illumination Variation assembly of phase-averaged and intensity-normalized images for each angle in SIM microscopy. The grey-white appearance of the output image indicates motion stability and evenness of the illumination, meaning that the movement of the chromosome is slow compared to the imaging acquisition time.

Supplementary Table 1. List of strains used in this study.

Strains	Descriptions	References
RRL189	AB1157, *ori1:: lacOx240::hygR, ter3::tetOx240::accC1, ΔgalK::tetR-mCerulean :: frt, ΔleuB::lacI-mCherry :: frt*	2
RRL150	AB1157, *ΔgalK::tetR-mCerulean :: aph frt, ΔleuB::lacI-mCherry :: cat frt*	2
RRL66	AB1157, *L3:: lacOx240::hygR, R3::tetOx240::accC1, ΔgalK::tetR-mCerulean :: frt, ΔleuB::lacI-mCherry :: frt*	3
FW1551	W3110, *hupA-mYPet :: aph frt*	4
REP1329	CM735, *dnaC2(ts) ΔmdoB::Tn10*	5
JW5794	BW25113, *ΔmdoB::aph frt*	6
JW3229-1	BW25113, *Δfis::aph frt*	6
JW1225-2	BW25113, *Δhns::aph frt*	6
JW0939	BW25113, *ΔmatP::aph frt*	6
JW5641	BW25113, *ΔslmA::aph frt*	6
JW3964	BW25113, *ΔhupA::aph frt*	6
JW0430	BW25113, *ΔhupB::aph frt*	6
FW1957	*dnaC2(ts) ΔmdoB::aph frt*	7
FW2177	AB1157, *ori1:: lacOx240::hygR, ter3::tetOx240::accC1, ΔgalK::tetR-mCerulean :: frt, ΔleuB::lacI-mCherry :: frt, hupA-mYPet :: frt*	7
FW2179	AB1157, *ori1:: lacOx240::hygR, ter3::tetOx240::accC1, ΔgalK::tetR-mCerulean :: frt, ΔleuB::lacI-mCherry :: frt, hupA-mYPet :: frt, dnaC2 (ts) :: aph frt*	7
FW2254	AB1157, *ori1:: lacOx240::hygR, ter3::tetOx240::accC1, ΔgalK::tetR-mCerulean :: frt, ΔleuB::lacI-mCherry :: frt, hupA-mYPet :: frt, ΔmatP::frt, dnaC2 (ts) :: aph frt*	7
FW2444	AB1157, *ori1:: lacOx240::hygR, ter3::tetOx240::accC1, ΔgalK::tetR-mCerulean :: frt, ΔleuB::lacI-mCherry :: frt, hupA-mYPet :: frt, Δfis::frt, dnaC2 (ts) :: aph frt*	7
FW2479	AB1157, *ori1:: lacOx240::hygR, ter3::tetOx240::accC1, ΔgalK::tetR-mCerulean :: frt, ΔleuB::lacI-mCherry :: frt, hupA-mYPet :: frt, Δhns::frt, dnaC2 (ts) :: aph frt*	7
FW2561	W3110, *hns-mYpet::aph frt*	This work
FW2564	W3110, *fis-mYpet::aph frt*	This work
FW2612	AB1157, *ori1:: lacOx240::hygR, ter3::tetOx240::accC1, ΔgalK::tetR-mCerulean :: frt, ΔleuB::lacI-mCherry :: frt, fis-mYPet :: aph frt, dnaC2 (ts) :: aph frt*	This work
FW2614	AB1157, *ori1:: lacOx240::hygR, ter3::tetOx240::accC1, ΔgalK::tetR-mCerulean :: frt, ΔleuB::lacI-mCherry :: frt, hns-mYPet :: aph frt, dnaC2 (ts) :: aph frt*	This work
FW2698	AB1157, *L3:: lacOx240::hygR, R3::tetOx240::accC1, ΔgalK::tetR-mCerulean :: frt, ΔleuB::lacI-mCherry :: frt*	This work
FW2721	*AB1157, ori1:: lacOx240::cat frt, R3::TetOx240::gmR, GalK::tetR-mCerulean::frt, Ori1::lacOx240::frt, LeuB::lacI-mCherry::frt, hupA-mYpet::frt DnaC2(ts) mdoB:: aph frt*	This work
FW2767	AB1157, *ori1:: lacOx240::hygR, ter3::tetOx240::accC1 ΔgalK::tetR-mCerulean :: frt, ΔleuB::lacI-mCherry :: frt, fis-mYPet :: aph frt, ΔhupA::frt, ΔhupB::frt, dnaC2 (ts) :: aph frt*	This work
AJ2830	MG1655, *hupA-mYPet :: frt, dnaC2 (ts) :: aph frt*	This work
AJ2836	AB1157, *hupA-mYPet :: frt, dnaC2 (ts) :: aph frt*	This work
JW1	AB1157, *ori1:: lacOx240::hygR, ter3::tetOx240::accC1 ΔgalK::tetR-mCerulean :: frt, ΔleuB::lacI-mCherry :: frt, hupA-mYPet :: frt, pdCas9rna3*	This work

Supplementary References

1. Saifi, B. & Ferat, J.-L. Replication Fork Reactivation in a *dnaC2* Mutant at Non-Permissive Temperature in *Escherichia coli*. *PLoS ONE* **7**, e33613, doi:10.1371/journal.pone.0033613 (2012).

2. Reyes-Lamothe, R., Possoz, C., Danilova, O. & Sherratt, D. J. Independent Positioning and Action of Escherichia coli Replisomes in Live Cells. *Cell* **133**, 90-102 (2008).

3. Wang, X., Liu, X., Possoz, C. & Sherratt, D. J. The two Escherichia coli chromosome arms locate to separate cell halves. *Genes Dev.* **20**, 1727-1731 (2006).

4. Datsenko, K. A. & Wanner, B. L. One-step inactivation of chromosomal genes in *Escherichia coli* K-12 using PCR products. *Proc. Natl. Acad. Sci. U.S.A.* **97**, 6640-6645, doi:10.1073/pnas.120163297 (2000).

5. Saifi, B. & Ferat, J.-L. Replication Fork Reactivation in a *dnaC2* Mutant at Non-Permissive Temperature in *Escherichia coli*. *PLoS ONE* **7**, e33613, doi:10.1371/journal.pone.0033613 (2012)

6. Baba, T. *et al.* Construction of Escherichia coli K-12 in-frame, single-gene knockout mutants: the Keio collection. *Mol. Syst. Biol.* **2**, 2006.0008, doi:10.1038/msb4100050 (2006).

7. Wu, F. *et al.* Cell boundary confinement sets the size and position of the E. coli chromosome. *bioRxiv*, doi.org/10.1101/348052 (2018).
