# Supplementary material for: Direct imaging of the circular chromosome in a live bacterium
Source: Nat Commun. 2019 May 16;10:2194. doi: 10.1038/s41467-019-10221-0 (PMC6522522; doi:10.1038/s41467-019-10221-0)
Supplement: Supplementary file 3 — Description of Additional Supplementary Files [file 41467_2019_10221_MOESM3_ESM.docx]

**Description of Supplementary Files**

**File Name:** Supplementary Video 1

**Description:** Dynamics of a circular chromosome imaged at 30 second time intervals using Structured Illumination Microscopy. Grey scale, HU-mYPet label. Red, Ori1 foci. Blue, Ter3 foci.

**File Name:** Supplementary Video 2

**Description:** Dynamics DNA spatial proximity maps derived from the time-lapse images of two single chromosomes shown in Video 1. The coordinates are the same as shown in Fig. 4B.
